# Effect of Eccentric Training with Different Durations, Intensities, and Contraction Velocities on Upper Limb Muscle Strength: A Meta-Analysis

**DOI:** 10.3390/life15030456

**Published:** 2025-03-13

**Authors:** Zhe Bai, Dong Zhang, Dongxue Liang, Xiaoke Chen, Xinyu Shi, Shu Chen

**Affiliations:** 1 Institute of Artificial Intelligence in Sports, Capital University of Physical Education and Sports, Emerging Interdisciplinary Platform for Medicine and Engineering in Sports, Beijing 100191, China; 19831306223@163.com (Z.B.); zhangdong2023@cupes.edu.cn (D.Z.); 13315316719@163.com (X.S.); 15293166839@163.com (S.C.); 2 Department of Physical Education, Tsinghua University, Beijing 100084, China; chenxk678@163.com

**Keywords:** eccentric training, upper limb muscle, muscle strength, intensity, duration, contraction velocity

## Abstract

Eccentric training may be more effective for muscle strength, but the optimal duration, intensity, and contraction velocity for improving upper limb strength remain unclear. We conducted a search across four databases (PubMed, Embase, Web of Science, and Cochrane) to evaluate the impact of eccentric training on upper limb skeletal muscle strength. A meta-analysis using standardized mean differences (SMDs) and 95% confidence intervals (CIs) was performed. The results from 11 studies involving 368 participants indicated that eccentric training significantly enhanced upper limb strength (SMD = 0.55, CI: 0.32–0.79). Subgroup analysis showed a slight increase in strength in the 1–4 week period (SMD = 0.38, CI: 0.02–0.73), a significant increase in the 4–8 week period (SMD = 0.69, CI: 0.27–1.10), and a substantial increase at 20 weeks (SMD = 0.71, CI: 0.22–1.21). Moderate intensity showed moderate improvements (SMD = 0.47, CI: 0.18–0.77), while high intensity led to significant adaptations (SMD = 0.70, CI: 0.30–1.10). Rapid eccentric contractions (SMD = 0.70, CI: 0.39–1.02) outperformed slow contractions (SMD = 0.35, CI: −0.01–0.71). Eccentric training is effective, with significant results generally requiring 4–8 weeks of high-intensity rapid eccentric training.

## 1. Introduction

Upper limb strength refers to the power of the muscles responsible for controlling the movement and stabilization of the shoulder, upper arm, forearm, and hand. These muscles are part of the appendicular system, which includes the bones and muscles that form the limbs. Specifically, upper limb strength involves key muscle groups such as those in the shoulder, upper arm, forearm, and hand/wrist [1]. The strength of the upper limb results from the coordinated action of these muscle groups, enabling both gross movements, like lifting and pushing, as well as fine motor control, such as grasping and manipulating objects [2]. These muscles are essential for a wide range of activities, from basic functional movements to sport-specific actions and intricate tasks like writing or typing.

Upper extremity muscle strength plays a critical role in athletic performance across a wide range of sports [3]. Strong upper limbs are vital for maintaining balance, stability, and efficient movement patterns [4]. For instance, while running, the upper body helps stabilize the trunk and lower limbs, reducing side-to-side swaying and improving the overall efficiency of movement [5]. Additionally, upper extremity strength is essential for sports that require rapid arm movements [6], such as throwing, swimming, boxing, and skiing. In throwing, the rapid extension of the arm and shoulder muscles is a key determinant of speed and distance [7], while in swimming, upper body strength and endurance significantly affect stroke mechanics and efficiency [8].

Resistance training is widely recognized as an effective strategy for improving upper body strength [9,10] and typically involves three types of muscle contractions: concentric, eccentric, and isometric [10]. All three types of muscle contractions generate muscle tension, which plays a central role in these processes. Muscle tension is the force produced within the muscle fibers when they are activated, whether through large contractions or muscle lengthening. Each type of contraction—concentric, eccentric, and isometric—produces distinct physiological effects. Concentric contractions occur when muscles shorten under load, eccentric contractions involve lengthening under tension, and isometric contractions involve no change in muscle length. While all forms of resistance training can enhance strength, they differ in their physiological mechanisms and outcomes. Among these, eccentric training has gained considerable attention due to its potential to generate higher peak forces and induce greater muscle hypertrophy compared to concentric and isometric training [11]. During eccentric contractions, muscles lengthen while under load, recruiting more motor units and activating type IIx muscle fibers, which are associated with greater strength and power [12]. This type of training results in greater muscle damage, driving more significant adaptations in the muscle’s cross-sectional area [13]. Compared to concentric contractions, eccentric actions typically involve more motor unit recruitment and faster cortical responses, leading to enhanced strength and power [14]. Additionally, eccentric training improves joint mobility and reduces metabolic cost [15]. The mechanisms underlying muscle hypertrophy in eccentric training are distinct from those of concentric or isometric training [16]. Eccentric contractions are particularly effective in improving explosive power by enhancing neural control and activating fast-twitch muscle fibers [17]. Furthermore, eccentric training enhances peak force production and induces broader neural adaptations, which contribute to greater overall muscle strength [18].

In practice, isolated eccentric movements are less common, but they can be effectively integrated into various exercise protocols [19]. Devices designed to optimize the eccentric phase of muscle contraction, such as flywheel training equipment, have become increasingly popular due to their ability to maximize training benefits [20]. Flywheel-type eccentric training uses a device where resistance is generated by the inertia of a flywheel, which is accelerated by the athlete’s own movement. Unlike isokinetic devices, where velocity is controlled by the machine, the velocity in flywheel training is determined by the athlete’s effort. Faster movements increase the resistance, providing a variable load that enhances both high-velocity and high-load eccentric contractions, which are key for building muscle strength and power [13]. This eccentric overload approach has been shown to enhance strength and hypertrophy more efficiently than traditional resistance training methods [21]. Flywheels, along with other specialized eccentric machines, offer a distinct advantage by intensifying the tension placed on muscles during the eccentric phase, which has been linked to superior muscle growth and strength adaptations [13,18,22].

While the impact of eccentric training on lower limb strength is well documented, with studies highlighting significant improvements in rapid power output and athletic performance [23], its application to upper limb strength remains underexplored. Given the importance of upper extremity strength in a wide range of sports, investigating the specific effects of eccentric training on upper body performance is of critical importance. Moreover, a detailed quantification of the effects of different types of eccentric training on upper limb strength is lacking. The types include different intervention periods, varying contraction intensities, and different contraction velocities, and their effects on strength adaptations. Understanding these variables can help in tailoring more effective training programs for athletes and practitioners seeking to optimize upper limb strength.

This meta-analysis aims to bridge this gap by evaluating existing studies on eccentric training and its impact on upper limb strength. By synthesizing data from a range of studies, this work will provide new insights into how eccentric training, with varying intervention periods, contraction velocities, and intensities, can be optimized to enhance upper body strength. It will offer both theoretical and practical implications for athletes and practitioners.

## 2. Materials and Methods

### 2.1. Search Strategy

The systematic review was conducted in accordance with the four stages outlined in the PRISMA guidelines: identification, screening, eligibility assessment, and inclusion. A comprehensive search was performed in the PubMed, Embase, Web of Science, and Cochrane databases up to 10 July 2024 to identify studies investigating the effects of eccentric exercise on the muscle strength of upper limb skeletal muscles. Only studies published in English were included. The primary search terms were “centrifugal training OR centrifugal exercise OR eccentric exercise OR eccentric training AND upper extremity OR upper extremities AND skeletal muscles OR muscle AND muscle tissue OR muscle strength AND athletes OR professional athletes OR elite athletes AND randomized controlled trial OR randomized OR placebo”.

### 2.2. Study Selection

Two reviewers (ZB and SC) initially removed duplicates using EndNoteX8 software. They then independently screened the titles and abstracts to identify potentially relevant studies, resolving any discrepancies through discussion with a third reviewer (XYS). Studies that met the inclusion criteria were independently identified and assessed by the same two reviewers. The specific inclusion criteria were as follows: (1) participants must be healthy adults, defined as individuals aged 18 to 65 years with no significant medical conditions or disabilities; (2) the intervention must involve any form of eccentric training; (3) the control group must either undergo the same form of concentric training or remain inactive during the experimental period; (4) studies must report at least one of the following outcomes: muscle strength, muscle morphology, or muscle mass; and (5) the study design must include randomized or quasi-randomized controlled trials or intervention-based experiments. Studies were excluded for the following reasons: (1) participants had any pathological conditions, (2) no form of eccentric training was applied, or (3) the study was not written in English.

### 2.3. Data Extraction

Two reviewers (ZB and SC) independently extracted data from the included studies, with any discrepancies resolved through discussion with a third reviewer (XYS). The extracted data encompassed study characteristics (first author’s name, publication year, and country of origin), participant characteristics (gender ratio and average age), intervention details (velocity of exercise, exercise intensity, training methods, intervention duration, and supervision status), and study outcomes (outcome measures, analysis methods, and units of measurement).

### 2.4. Quality Assessment

The risk of bias was assessed by two pairs of authors (ZB and SC; XYS and ZB) using the Cochrane Collaboration’s risk-of-bias tool [24]. Given the nature of exercise interventions, blinding participants in the included studies is impractical; therefore, only six other types of bias risk were evaluated, including random sequence generation, allocation concealment, blinding of outcome assessors, incomplete outcome data, selective outcome reporting, and other risks of bias.

### 2.5. Sensitivity Analysis

This study originally included thirteen primary studies, and after conducting a sensitivity analysis, two studies that contributed to high overall heterogeneity were excluded: before exclusion [SMD = 0.13, 95% CI: −0.39 to 0.65, *p* < 0.00001, I^2^ = 81%] and after exclusion [SMD = 0.55, 95% CI: 0.32 to 0.79, *p* < 0.00001, I^2^ = 0%]. The high overall heterogeneity caused by these two studies is speculated to be due to several factors. One study had an extremely short intervention period, resembling the results of a single experiment, focusing more on muscle hardening and injury mechanisms [25]; the other study had an excessively long interval between interventions, with a 4-week gap between each exercise session. This interval may not have produced significant training effects and also explored adverse physical reactions using eccentric training, which differs from other studies where eccentric training aims primarily at increasing muscle strength [26]. As a result, there may be significant differences in outcome indicators and training effects when compared to other studies in the literature.

### 2.6. Statistical Analysis

Data extraction involved calculating the changes in mean and standard deviation (SD) before and after the intervention. The research sample data were input into the Review Manager 5.3 software. Due to the use of different units in reporting among the original studies, standardized mean differences (SMDs) and 95% confidence intervals (CIs) were employed and calculated using a fixed-effect model. After determining the overall effect size, subgroup analyses were conducted based on the intervention period (1–4 weeks, 4–8 weeks, and 20 weeks), training intensity (moderate intensity and high intensity), and contraction velocity (fast eccentric training and slow eccentric training). Training intensity was clearly defined using the percentage of 1RM, with moderate intensity ranging from 60 to 75% 1RM and high intensity ranging from 75 to 90% 1RM. Since the interventions in the included studies involved both isokinetic dynamometers and other non-isokinetic resistance equipment, the criteria for classifying contraction velocity were as follows: fast eccentric training was defined as an isokinetic angular velocity greater than or equal to 30°/s or training intensity between 75 and 90% 1RM, while slow eccentric training was defined as an isokinetic angular velocity less than 30°/s or training intensity between 60 and 75% 1RM. The significance level was set at *p* < 0.05. Heterogeneity was quantified using the I^2^ statistic. The Cochrane guidelines were used to interpret I^2^ as follows: 25% indicating low heterogeneity, 50% indicating moderate heterogeneity, and 75% indicating high heterogeneity. Publications that contributed to high heterogeneity were excluded through a sensitivity analysis. A funnel plot was utilized to assess publication bias.

## 3. Results

### 3.1. Included Studies

**Figure 1:**
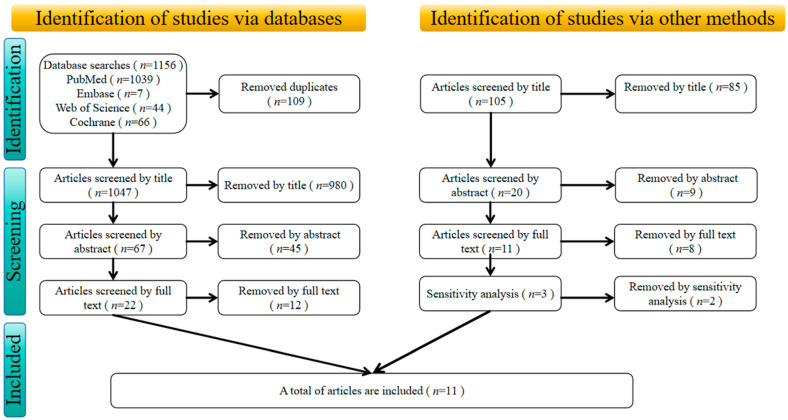
The flowchart illustrates the process of the literature search and screening.

After conducting a comprehensive search of multiple databases, a total of 1156 articles were identified (PubMed: 1039; Embase: 7; Web of Science: 44; Cochrane: 66), with an additional 105 articles sourced from other origins. Following the removal of 109 duplicate documents, 1065 articles were screened based on their titles. A further 54 articles were excluded after abstract review, leaving 33 articles for full-text assessment. Of these, 20 were excluded for the following primary reasons: lack of centrifugal training (*n* = 5), interventions targeting the lower body (*n* = 9), subjects in an inappropriate state (*n* = 6), and exclusion due to sensitivity analysis (*n* = 2). Ultimately, 11 articles met the inclusion criteria, as shown in Figure 1.

### 3.2. Description of the Included Trials

Table 1 outlines the main characteristics of the included studies, including participant demographics, interventions, and outcome measures. A total of 368 participants were involved, with 206 individuals performing eccentric exercises and the remaining participants serving as a control group or engaging in alternative exercise forms. The gender distribution was uneven, with 108 males and 80 females in the eccentric group, and the gender of 26 participants was undisclosed. Training loads were applied using isokinetic eccentric dynamometers in seven studies and resistance equipment in four studies. The average training duration was 6.3 ± 4.9 weeks (ranging from 1 to 20 weeks), with a weekly frequency of 2.4 ± 1.1 sessions (ranging from 1 to 5 sessions) and an average of 8.1 ± 4.9 repetitions per session (ranging from 1 to 20 repetitions). Training intensity was generally moderate to high, with all interventions conducted under supervision. The intervention period ranged from 1 to 20 weeks, with weekly intervention frequencies ranging from two to five sessions. However, the specific weekly intervention frequency was not disclosed in the study by Nickols [27]. Muscle strength was the outcome measure in all studies, and the analysis method employed was an analysis of variance (ANOVA).

### 3.3. Quality Assessment

**Figure 2:**
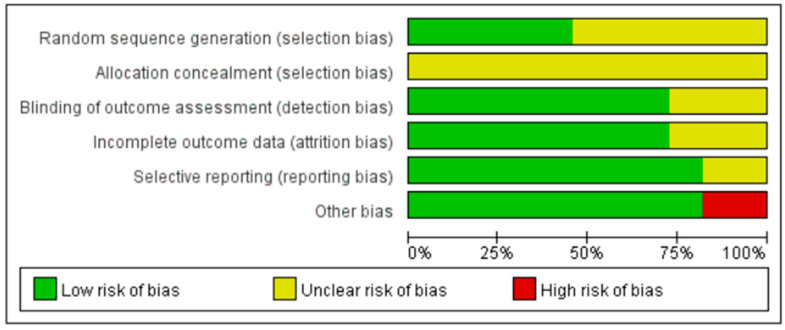
Overall quality assessment diagram of studies included in the research.

Figure 2 summarizes the quality assessment for each study, highlighting specific risks of bias. Notably, two articles have a sample size below 10 participants, which raises concerns about the reliability of their outcome indicators due to the small number of subjects.

### 3.4. Synthesis of the Results

**Figure 3:**
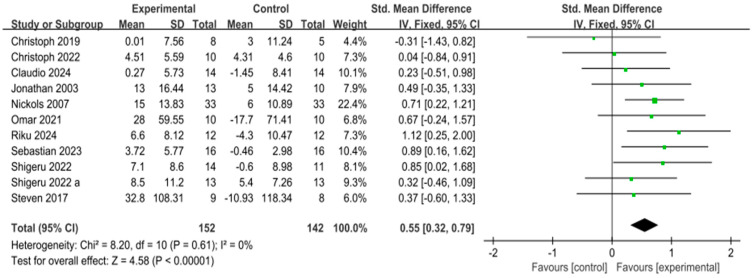
Forest plot of muscle strength changes after eccentric training [9,10,11,22,25,26,27,28,29,30,31,32,33]. The “a” in Shigeru 2022 a is used to distinguish it from another article, Shigeru 2022, which was published in the same year by the same author. In the figure, the green dots represent the effect size of individual studies, while the black squares below represent the estimated effect size of individual studies. The size of the black squares is proportional to the weight of the study, with larger squares indicating studies with greater weight.

**Figure 4:**
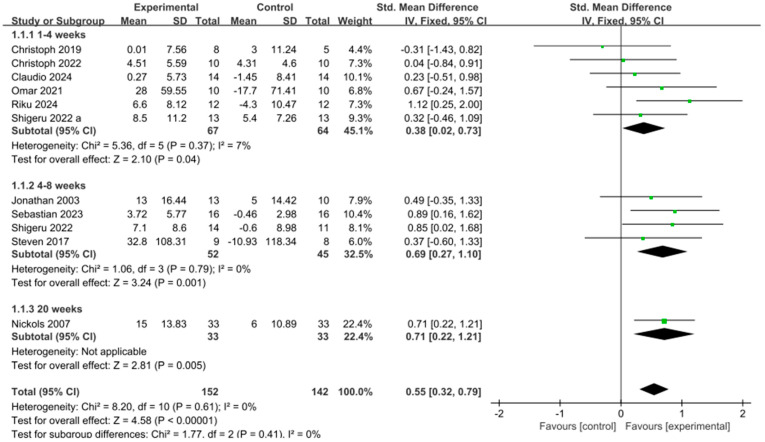
Subgroup forest plot of muscle strength changes after eccentric training, summarizing training periods of 1–4 weeks, 4–8 weeks, and 20 weeks [9,10,11,22,25,26,27,28,29,30,31,32,33]. The “a” in Shigeru 2022 a is used to distinguish it from another article, Shigeru 2022, which was published in the same year by the same author. In the figure, the green dots represent the effect size of individual studies, while the black squares below represent the estimated effect size of individual studies. The size of the black squares is proportional to the weight of the study, with larger squares indicating studies with greater weight.

Based on 11 experimental groups, eccentric training was found to have a significant positive effect on muscle strength compared to the control group [SMD = 0.55, 95% CI: 0.32 to 0.79, *p* < 0.00001], with no heterogeneity across the studies (I^2^ = 0%), as shown in Figure 3. Subgroup analysis of the intervention period revealed that eccentric training for 4–8 weeks [SMD = 0.69, 95% CI: 0.27 to 1.10, *p* = 0.001, I^2^ = 0%] and for 20 weeks [SMD = 0.71, 95% CI: 0.22 to 1.21, *p* = 0.005, I^2^ = 0%] had a significant positive effect on the increase in upper limb strength. The intervention period of 1–4 weeks [SMD = 0.38, 95% CI: 0.02 to 0.73, *p* = 0.04, I^2^ = 7%] also showed a positive effect, although it was less pronounced compared to the longer intervention periods, as seen in Figure 4.

**Figure 5:**
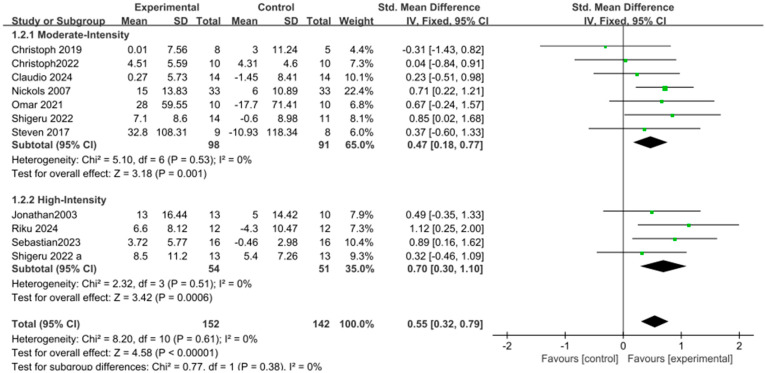
Subgroup forest plot of muscle strength changes after eccentric training, summarizing moderate-intensity and high-intensity training methods [9,10,11,22,25,26,27,28,29,30,31,32,33]. The “a” in Shigeru 2022 a is used to distinguish it from another article, Shigeru 2022, which was published in the same year by the same author. In the figure, the green dots represent the effect size of individual studies, while the black squares below represent the estimated effect size of individual studies. The size of the black squares is proportional to the weight of the study, with larger squares indicating studies with greater weight.

Additionally, subgroup analysis of intervention intensity revealed significant differences in the effect on upper limb strength between moderate-intensity [SMD = 0.47, 95% CI: 0.18 to 0.77, *p* = 0.001, I^2^ = 0%] and high-intensity [SMD = 0.70, 95% CI: 0.30 to 1.10, *p* = 0.0006, I^2^ = 0%] eccentric interventions, with high-intensity training demonstrating a more pronounced effect compared to moderate-intensity training, as shown in Figure 5.

**Figure 6:**
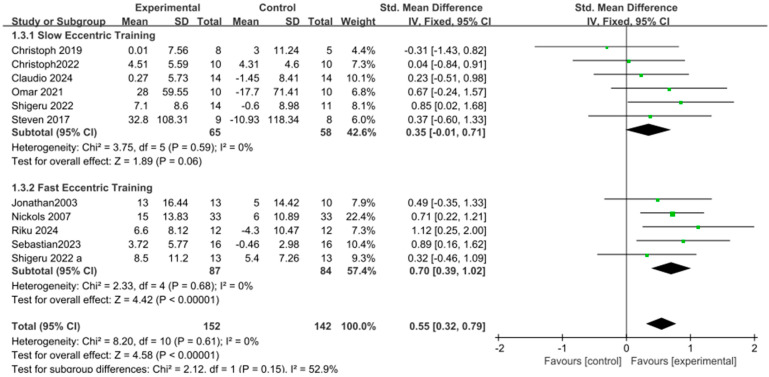
Subgroup forest plot of muscle strength changes following eccentric training, categorized by slow and fast eccentric contraction velocities [9,10,11,22,25,26,27,28,29,30,31,32,33]. The “a” in Shigeru 2022 a is used to distinguish it from another article, Shigeru 2022, which was published in the same year by the same author. In the figure, the green dots represent the effect size of individual studies, while the black squares below represent the estimated effect size of individual studies. The size of the black squares is proportional to the weight of the study, with larger squares indicating studies with greater weight.

Finally, the subgroup analysis of contraction velocity showed a significant difference in the effect on upper limb strength between slow eccentric contractions [SMD = 0.35, 95% CI: −0.01 to 0.71, *p* = 0.06, I^2^ = 0%] and fast eccentric contractions [SMD = 0.70, 95% CI: 0.39 to 1.02, *p* < 0.00001, I^2^ = 0%]. Fast eccentric contractions had a more pronounced promoting effect on upper limb strength compared to slow eccentric contractions, as shown in Figure 6.

## 4. Discussion

### 4.1. Main Findings

In most sports, athletes rely on the fine motor control and flexibility of their upper limbs, which are crucial for technical execution and injury prevention [34]. The foundation of these abilities lies in having a strong base of strength, which supports optimal movement control and helps prevent injury [35]. Eccentric training is undoubtedly an effective method for enhancing strength. Previous studies have examined the effects of eccentric training on the shoulder joint [36], elbow joint [37], and the entire upper limb [20], all of which align with the findings of this study, showing positive benefits. This study aims to summarize these intervention methods and explore more effective training programs from different perspectives. The article reviews the impact of eccentric training on upper limb strength, based on 11 studies involving 368 participants and various eccentric intervention techniques. The conclusion affirms the positive effect of eccentric training in increasing upper limb strength. Subgroup analyses confirmed that training interventions of 4–8 weeks and 20 weeks significantly improve muscle strength, while effects within 1–4 weeks are relatively less pronounced. Both moderate and high-intensity training interventions can significantly enhance muscle strength, and rapid eccentric contractions notably improve muscle strength, whereas slow eccentric contractions show less significant effects. The multiple studies included in this meta-analysis provide comprehensive theoretical support.

Due to the diverse intervention methods and approaches in the original studies included in the research, it is not possible to determine which method is the most effective. As a result, the article conducted a subgroup analysis. The analysis of the intervention period revealed no significant difference between interventions lasting 4–8 weeks and those lasting 20 weeks, with both demonstrating a clear effect on enhancing upper limb strength. However, the intervention effect during the 1–4-week period was relatively less pronounced compared to the longer durations. Previous studies have suggested that early increases in the muscle’s cross-sectional area may be attributed to edema [14], indicating that true strength adaptations may require a longer intervention period to be fully observed. Based on the results of this study, if the goal is to enhance strength through eccentric training, short-term interventions are less effective, and it is recommended to extend the intervention period to more than 4–8 weeks. For both athletes and recreational exercisers, this implies that achieving meaningful strength capacity adaptations from eccentric training necessitates consistent, longer-term commitment, particularly for those aiming to enhance performance in strength-dependent sports or to improve overall functional strength.

Like any study, the current research has limitations that must be taken into account when interpreting the results. One limitation is that the data were pooled from different measurement units—such as g/kg, percentages, mm, cm, L, and cm^3^—as well as from various measurement methods [14]. To address this, the article conducted an additional subgroup analysis. The analysis of training intensity revealed that both moderate and high training intensities have a significant effect on upper limb strength, with no heterogeneity observed. Moreover, high-intensity training proved to be relatively more effective. These findings align with previous research on eccentric training interventions in elderly individuals [19,38]. Combining the results of the two subgroups, it can be concluded that high-intensity eccentric training lasting more than 4–8 weeks is a more effective program for increasing upper limb muscle strength.

Previous studies have shown that eccentric exercise is more effective than other forms of contraction in promoting strength adaptations [39], but these increases are highly dependent on the mode of contraction and the velocity of movement [40,41]. Contractions at different velocities have distinct effects on muscles [42]. Therefore, this study conducted a subgroup analysis comparing rapid eccentric training and slow eccentric training. The results indicate that rapid eccentric contractions produce relatively better effects, while slow eccentric contractions show less significant outcomes. Combining the results from the three subgroups, it can be concluded that high-intensity rapid eccentric training lasting more than 4–8 weeks is an effective training regimen for increasing upper limb strength.

For athletes and sports enthusiasts, particularly those involved in sports that heavily rely on upper body strength—such as swimming, baseball, tennis, and basketball—eccentric training is an effective strategy for enhancing both strength and power output [43]. Improvements in eccentric strength contribute to better muscle control, injury prevention, and enhanced technical performance [44]. As research suggests, the most effective training programs for increasing upper body strength involve at least 4 weeks of training, with maximum benefits typically appearing after 8 weeks or longer. Specifically, athletes should consider high-intensity eccentric training, as this meta-analysis demonstrates that high-intensity eccentric training is more effective than moderate-intensity training. High-intensity eccentric training offers several benefits, including greater muscle fiber recruitment, increased muscle size, and improved neuromuscular efficiency [44]. This method also enhances tendon stiffness and muscle elasticity, which helps prevent injuries such as tendinitis, rotator cuff tears, and ligament strains—common injuries in activities involving rapid or high-velocity movements [45]. Furthermore, the increased muscle force generated by eccentric contractions can lead to improved performance metrics, such as throwing speed, sprinting velocity, and jump height [46]. Additionally, compared to slower eccentric contractions, faster eccentric contractions—those involving quicker muscle elongation under load—have been shown to result in more significant strength adaptations. This is particularly beneficial for athletes in power and speed-based sports (e.g., sprinting or basketball), where rapid deceleration and reactivity are crucial. By incorporating fast eccentric movements into training, athletes can enhance their deceleration capacity, improve overall performance, and reduce the risk of injuries caused by sudden movements or changes in direction [47].

Finally, it is important to recognize the limitations of this study. Although subgroup analyses were conducted from three different perspectives, external factors such as age, gender, and testing equipment could still influence heterogeneity or experimental outcomes. Additionally, this study did not include non-English articles, which may have led to the exclusion of relevant studies in the literature. Regarding the subgroup analysis of duration, the training frequency varied across studies, potentially contributing to discrepancies in the results. While the validity of these findings requires further confirmation in these areas, the results still support the effectiveness of this exercise regimen, offering valuable guidance for coaches and athletes in various sports.

### 4.2. Training Implications

In this study, we found that eccentric training significantly enhanced upper limb strength. This finding aligns with previous research, such as [18], which demonstrated that eccentric training can lead to notable strength adaptations in various populations. Through subgroup analysis, we observed a clear trend in upper body strength improvements as the training period increased. Specifically, 1–4 weeks of training resulted in modest strength improvements, while 4–8 weeks and 20 weeks of training led to significantly greater adaptations in strength, similar to findings by [41], who reported enhanced strength following longer training periods.

Additionally, we observed that the intensity of eccentric training plays a critical role in strength development. Moderate-intensity training led to moderate strength adaptations, while high-intensity training resulted in substantial strength improvements, which is consistent with findings by [42], who noted that higher-intensity eccentric training yields more significant strength outcomes.

The subgroup analysis, which classified eccentric contractions into slow and fast velocities, revealed a significant difference between the two methods, with fast eccentric contractions leading to greater strength adaptations compared to slow contractions. This supports the findings of [43], who suggested that fast eccentric movements can more effectively enhance power output. Based on these findings, we propose the following recommendations for applying eccentric training to different populations:

Training Duration and Frequency:

It is recommended to incorporate eccentric training into a long-term training program that lasts at least 4–8 weeks for individuals aiming for moderate strength adaptations. For those seeking to maximize strength increases, a 20-week training cycle may be ideal. These durations are supported by the findings of [44], who observed optimal tendon adaptation and strength adaptations after prolonged training periods of similar length.

Intensity Adjustments Based on Fitness Levels:

The intensity of eccentric training should be adjusted according to personal training goals and fitness levels. For individuals who are newer to strength training or those seeking moderate strength adaptations, a moderate-intensity program may be more suitable. For athletes or experienced fitness enthusiasts looking for significant strength improvements, high-intensity eccentric training is recommended. This approach is consistent with recommendations in the literature, such as those from [29,32,45], which emphasize the importance of intensity in achieving greater muscular adaptations.

Contraction Velocity:

When choosing the contraction velocity, this study found a significant difference in upper limb strength adaptations between fast and slow eccentric contractions, with fast eccentric contractions being more effective for enhancing strength. Specific recommendations for fast eccentric training include the following: (1) Fast eccentric descent: For example, during bicep curls, the dumbbells should be quickly released to lower them, and then the concentric contraction should be swiftly controlled and completed. (2) Dynamic eccentric training: For example, during clapping push-ups, after clapping, the body should be controlled, and you should land with both hands supporting the body without cushioning downward. (3) Using high-velocity isokinetic muscle strength testing equipment for rapid, controlled eccentric training: For example, during flywheel training, flywheel training devices provide variable resistance during both concentric and eccentric phases, with eccentric loading being more pronounced. The flywheel mechanism harnesses inertia, enabling fast, controlled eccentric contractions. By accelerating the flywheel during the concentric phase and controlling its deceleration during the eccentric phase, these devices effectively replicate high-speed eccentric contractions common in athletic movements, such as sprinting or throwing. Flywheel training also supports progressive overload, as resistance increases with the speed of the flywheel.

Personalized Training Plans:

Considering individual differences, it is highly recommended that personalized training plans be formulated under the guidance of a professional trainer. These plans should account for individual conditions such as age, gender, fitness level, and specific sport requirements. For instance, elderly populations or individuals recovering from injury may benefit from a more moderate-intensity or lower-frequency training cycle to avoid injury while still enhancing strength, as noted in studies by [20].

Finally, we look forward to further research on the effectiveness of eccentric training across various populations, such as older adults and rehabilitation patients. Exploring how eccentric training can be integrated with other training methods, including concentric and isometric training, could offer comprehensive physical fitness benefits. Additionally, we are eager to investigate the potential of eccentric training to improve sports performance and prevent injuries. As research progresses, there is considerable potential for eccentric training to become an essential tool for enhancing upper body strength and promoting overall health across diverse populations.

## 5. Conclusions

This meta-analysis consolidates a wealth of existing research, highlighting the significant impact of eccentric training on enhancing upper limb strength. A robust effect size indicates that high-intensity, rapid eccentric training, conducted over a period of more than 4–8 weeks, is an effective method for improving strength in both the general adult population and athletes. These findings offer valuable insights for coaches and the athletic community. However, considering the identified limitations, further research is needed to confirm the generalizability of these results. Moving forward, it is essential to explore this outcome from diverse perspectives, including gender, physical capabilities, and equipment, in order to provide more comprehensive and direct evidence and refine the conclusions drawn from this study.

## Figures and Tables

**Table 1:** Characteristics of included studies.

Study	Characteristics of Subject		Intervention Information					Outcomes	
	Number (Male/Female)	Age (Mean ± SD)	Type of Exercise	Intensity	Duration of Intervention and Loads	Period and Frequency (Week × Sessions per Week)	Supervised or Nonsupervised	Outcomes	Unit
Jonathan (2003) [28]	4 M 9 F	21.9 ± 1.5	RC	high	2–6 sets of 8 reps at 75–90% 1RM 180°/s ECC and CON	8 × 3	supervised	muscle strength	NM
	7 M 6 F	19.4 ± 0.6	SC	high	2–6 sets of 8 reps at 75–90% 1RM 30°/s ECC and CON				
	2 M 8 F	22.7 ± 0.9	No exercise						
Nickols (2007) [27]	33 F	20.1 ± 1.4	ECC	moderate	5 sets of 6 reps at 60°/s ECC, 60–75% 1RM	20 weeks	supervised	muscle strength	NM
	37 F	20.2 ± 1.9	CON	moderate	5 sets of 6 reps at 60°/s CON, 60–75% 1RM				
Michael (2013) [26]	18 M	30.8 ± 1.2	ECC		10 sets of 6 reps at 1RM ECC	2 weeks	supervised	muscle strength, muscle hypertrophy	KG
Steven (2017) [29]	9	23 ± 3	ECC	moderate	1 set of 10 reps at 1RM 75% ECC	7 × 3	supervised	muscle srength	KG
	8	24 ± 4	CON	moderate	1 set of 10 reps at 1RM 75% CON				
Christoph (2019) [22]	9	21.4 ± 1.9	ECC	moderate	3–4 sets of 4 reps at 60–75% 1RM ECC	4 × 2	supervised	muscle strength	KG
Omar (2021) [10]	6 M 4 F	24.3 ± 3.5	ECC	moderate	3–6 sets of 10 reps at 60–75% 1RM ECC and CON	4 × 3	supervised	muscle strength	KG
	6 M 4 F		CON-ECC	moderate	3–6 sets of 10 reps at 60–75% 1RM ECC and CON				
	6 M 4 F		No exercise						
Shigeru (2022) [11]	7 M 7 F	21.1 ± 1.5	CON-ECC	moderate	3 sets of 10 reps at 60–75% 1RM 30°/s CON and 30°/s ECC	5 × 2	supervised	muscle strength, muscle hypertrophy	NM
	6 M 8 F	20.6 ± 1.0	CON	moderate	3 sets of 10 reps at 60–75% 1RM 30°/s CON				
	7 M 7 F	20.8 ± 1.0	ECC	moderate	3 sets of 10 reps at 60–75% 1RM 30°/s ECC				
	5 M 6 F	21.2 ± 0.6	No exercise						
Shigeru (2022) [30]	9 M 4 F	21.0 ± 0.9	MVC-ISO	high	1 set of 1 rep at 75–90% 1RM MVC-ISO	4 × 5	supervised	muscle strength	NM
	9 M 4 F	21.2 ± 0.7	MVC-ECC	high	1 set of 1 rep at 75–90% 1RM 30°/s ECC				
	10 M 3 F	21.4 ± 0.7	MVC-CON	high	1 set of 1 rep at 75–90% 1RM 30°/s CON				
	7 M 3 F	22.2 ± 0.7	No exercise						
Christoph (2022) [9]	10 M	22.1 ± 2.9	IC	moderate	2–3 sets of 4 reps at 60–75% 1RM ECC	6 × 2	supervised	muscle strength, strength endurance	KG
Mitsuyoshi (2022) [25]	10 M	25.8 ± 3.9	ECC, CON		5 sets of 10 reps at 1RM 50% ECC and CON	1 × 4	supervised	muscle hardness	KG
Sebastian (2023) [31]	16 M	23.3 ± 3.9	IC	high	5 sets of 10 reps at 75–90% 1RM 30, 60, and 180°/s ECC	6 × 2	supervised	muscular structure	NM
Riku (2024) [32]	8 M 4 F	21.4 ± 1.2	MVC-ECC	high	1 set of 6 reps at 75–90% 1RM MVC-ECC	4 weeks	supervised	muscle strength	NM
Claudio (2024) [33]	5 M 9 F	29.5 ± 2.5	ECC	moderate	20 reps at 60–75% 1RM ECC	1 × 3	supervised	muscle strength	KG

M, male; F, female; CON, concentric contraction; ECC, eccentric contraction; CON-ECC, concentric-eccentric; IC, isokinetic centrifugation; RC, rapid centrifugation; SC, slow centrifugation; NM, newton meter; KG, kilogram; MVC, maximum voluntary isometric contraction.

## Data Availability

The dataset is available upon request from the authors.
